# Clinical Characteristics and Management of Statin-Associated Anti-3-Hydroxy-3-Methylglutaryl-Coenzyme A Reductase Immune-Mediated Necrotizing Myopathy

**DOI:** 10.3390/jcm14186610

**Published:** 2025-09-19

**Authors:** Jiyeol Yoon, Seung Woo Kim, Se Hoon Kim, Jason Jungsik Song, Yong-Beom Park, Hee Jin Park, Ha Young Shin, Se Hee Park, Yumie Rhee

**Affiliations:** 1 Division of Rheumatology, Department of Internal Medicine, Yonsei University College of Medicine, Seoul 03722, Republic of Korea; jiyoonmd@yuhs.ac (J.Y.); jsksong@yuhs.ac (J.J.S.); yongbpark@yuhs.ac (Y.-B.P.); phj0111@yuhs.ac (H.J.P.); 2 Department of Neurology, Severance Hospital, Yonsei University College of Medicine, Seoul 03722, Republic of Korea; kswneuro@yuhs.ac (S.W.K.); hayshin@yuhs.ac (H.Y.S.); 3 Department of Pathology, Yonsei University College of Medicine, Seoul, Republic of Korea; paxco@yuhs.ac; 4 Institute for Immunology and Immunological Diseases, Yonsei University College of Medicine, Seoul 03722, Republic of Korea; 5 Department of Internal Medicine, Institute of Endocrine Research, Yonsei University College of Medicine, Seoul 03722, Republic of Korea; shpark528@yuhs.ac

**Keywords:** autoimmune myopathy, statin, anti-HMGCR, immune-mediated necrotizing myopathy

## Abstract

**Background**: Immune-mediated necrotizing myopathy (IMNM) associated with anti-3-hydroxy-3-methylglutaryl-coenzyme A reductase (HMGCR) antibody is a rare but critical complication usually triggered by statin use. However, the comprehensive characterization and long-term outcomes of anti-HMGCR-positive IMNM remain underexplored. This study aimed to examine the clinical characteristics, diagnostic challenges, treatment responses, and long-term outcomes of patients with anti-HMGCR-positive IMNM. **Methods**: A retrospective review was conducted at a single institution between 2019 and 2025 to analyze the data of patients diagnosed with anti-HMGCR-positive IMNM. Diagnoses were confirmed by detecting anti-HMGCR antibodies and meeting the criteria for IMNM of the European Neuromuscular Center. The analyzed data included demographics, clinical presentation, laboratory findings, imaging results, muscle biopsy characteristics, treatment regimens, and follow-up outcomes. **Results**: Ten patients (six women and four men) with a median age of 58 (range, 33–86) years were included. Nine patients had a history of statin use for a median duration of two years. The average diagnostic delay was 233 days after the onset of symptoms. The initial creatine kinase (CK) levels ranged from 1438 to over 13,000 IU/L. Muscle biopsies revealed necrosis and regeneration of muscle fibers. CK levels fluctuated and trended downward over 180 days post-treatment. Treatment included corticosteroids, methotrexate, azathioprine, tacrolimus, mycophenolate, intravenous immunoglobulin, and rituximab. Delayed treatment initiation from symptom onset was correlated with prolonged treatment time until the first remission. **Conclusions**: The prognosis of anti-HMGCR-positive IMNM is less favorable when treatment is delayed after symptom onset. Further research is warranted to identify poor prognostic markers and develop relevant treatments.

## 1. Introduction

Statin administration can cause myalgia and elevated muscle enzymes [1]. However, it is partially established that certain individuals develop an autoimmune response against 3-hydroxy-3-methylglutaryl-coenzyme A reductase (HMGCR), the target enzyme of statins, leading to a distinct form of immune-mediated necrotizing myopathy (IMNM) [2]. IMNM is a clinically distinct subset of idiopathic inflammatory myopathies characterized by muscle necrosis, elevated creatine kinase (CK) levels, and specific autoantibodies [3,4]. IMNM histology exhibits prominent muscle fiber necrosis and myophagocytosis with minimal lymphocytic infiltration [5]. Anti-HMGCR-positive IMNM represents a subset of IMNM, which also includes anti-signal recognition particle (SRP) positive IMNM and seronegative IMNM, the latter of which lacks both anti-HMGCR and anti-SRP antibodies [6]. While anti-SRP-positive IMNM is associated with cardiomyopathy as an extramuscular manifestation, both anti-HMGCR-positive IMNM and seronegative IMNM may be linked to malignancy. Anti-HMGCR-positive myopathy was recognized as a novel form of IMNM a decade ago [7]. The prevailing hypothesis is that individuals with specific immunological predispositions may develop anti-HMGCR antibodies following a triggering event such as myopathy or muscle damage. This event, coupled with the overexpression of the HMGCR antigen induced by statins, may amplify the immune response against the HMGCR enzyme, which plays a crucial role in cellular metabolism [4,8]. Anti-HMGCR autoantibodies specifically target the intracellular C-terminus of the enzyme, exerting a pathogenic effect on skeletal muscle cells and indirectly disrupting the cholesterol synthesis pathway [9,10]. Clinical and experimental evidence suggest that anti-HMGCR antibodies are directly involved in the pathogenesis of IMNM, leading to muscle fiber damage and necrosis [3,11].

IMNM’s incidence has rarely been assessed because of clinical negligence and under-recognition of the disease. It is classified within the spectrum of idiopathic inflammatory myopathies, which also include dermatomyositis, polymyositis, antisynthetase syndrome, and inclusion body myositis [4]. The estimated incidence of inflammatory myopathies is 1.16–19 per million per year, with a prevalence of 2.4–33.8 per 100,000 individuals [12]. The incidence of anti-HMGCR-positive IMNM is thought to be 2 per million person-years [13,14]. Nonetheless, the true prevalence and incidence of underestimated IMNM might be challenging to determine, as they could represent a substantial portion.

Therefore, we aimed to present a case series of ten patients with anti-HMGCR-positive IMNM and describe their clinical characteristics, prognostic factors, treatment responses, and long-term outcomes, along with a literature review, to benefit clinical settings and emphasize the significance of this emerging complication of statin use. This case series aims to enhance clinical knowledge of this distinct myopathy and provide insights into its optimal management strategies.

## 2. Materials and Methods

### 2.1. Patients and Data Collection

We examined the electronic medical records of all patients treated for IMNM in the Neurology and Rheumatology Departments to identify the presence of anti-HMGCR antibodies in their sera. After identifying patients diagnosed with anti-HMGCR-positive IMNM at our facility between 2018 and 2023 using the European Neuromuscular Center (ENMC) criteria for IMNM [15], we retrospectively reviewed the clinical data of the enrolled patients. Elevated serum CK levels, proximal muscle weakness, and the presence of anti-HMGCR autoantibodies are the diagnostic criteria for anti-HMGCR-positive IMNM. Furthermore, we defined and recruited possible anti-HMGCR-positive IMNM patients as those with anti-HMGCR antibody presence and elevated serum CK levels with normal muscle function, regardless of normal or absent muscle biopsy results [16]. We obtained demographic, clinical, and laboratory data from patient records, along with non-invasive and invasive assessments, including electromyography, nerve conduction studies, magnetic resonance imaging (MRI), and positron emission tomography-computed tomography (PET-CT). Finally, we evaluated the treatments and outcomes. Additionally, all patients were assessed for past and current use of statins. Muscle biopsies were analyzed by experienced neuropathologists, focusing on necrosis, regeneration, inflammation, and major histocompatibility complex (MHC) class I expression, as well as C5b9 (membrane attack complex of complement, MAC), which is crucial for distinguishing IMNM from other myopathies [3].

### 2.2. HMGCR Antibody Testing

Serum samples were analyzed for anti-HMGCR antibodies using the chemiluminescence QUANTA Flash^®^ HMGCR assay (Werfen Inc., Barcelona, Spain), which involves recombinant human HMGCR antigen attached to paramagnetic beads within the BIO-FLASH system^®^ (Werfen Inc., Barcelona, Spain). A positive result was indicated by a value of >20 chemiluminescence units (CU). Testing was conducted by Seoul Clinical Laboratories (SCL Inc., Gyeonggi-do, Korea), a certified diagnostic facility that ensured precise and reliable detection of antibodies.

### 2.3. Immunohistochemical Study

Open muscle biopsies were mainly performed on the rectus femoris, vastus lateralis, and deltoid muscles of the patients. The muscle tissue was routinely frozen in isopentane cooled with liquid nitrogen and stored at −70 °C. Routine staining techniques included hematoxylin and eosin (H&E), modified Gomori trichrome, and enzyme histochemistry for mitochondria. Immunoperoxidase staining for MHC-I, MHC-II, CD45, CD68, and C5b9 MAC was performed on 8-μm acetone-fixed cryostat sections using the streptavidin-biotin complex technique, with diaminobenzidine as a color indicator. Immunostaining for T- and B-cell subsets was performed on paraffin sections using the same technique.

### 2.4. Outcome Data

To assess the treatment outcomes, we collected data on the Medical Research Council muscle strength grades for upper arm abduction, lower leg hip flexion, and overall evaluation using the Walton-Gardner-Medwin scale [17] (Table 1). Muscle strength was rated on a scale of 0–5, with 0 indicating no strength and 5 denoting full strength against resistance. In addition to muscle strength, serum CK levels were recorded at initial presentation and during follow-up. Although patients usually show improved muscle strength with decreased CK levels during treatment, they rarely achieve or maintain normal CK levels during follow-up. Therefore, we defined complete remission as CK levels below 500 IU/L and stable muscle strength without newly developed weakness or worsening of previously improved conditions after treatment [16]. Conversely, relapse was defined as an increase in muscle enzyme levels above 500 IU/L, with or without a decrease in muscle strength.

### 2.5. Ethics

The Severance Hospital Institutional Review Board (IRB; 4-2025-0502) granted approval for this study. Due to the retrospective nature of the study and the use of anonymized patient data, the IRB waived the requirement for informed consent.

### 2.6. Statistical Analysis

Continuous variables were reported as mean ± standard deviation (SD) and median. Categorical variables were expressed as numbers and percentages. Spearman’s rho was calculated to assess the correlation between the initial creatine kinase (CK) level and the quantitative measurement of anti-HMGCR antibody, as well as between the time span of delayed treatment administration and the time required to achieve remission. The regression line was illustrated in the scatter plot. To compare treatment responses, we plotted the serum CK levels over the observation time following treatment initiation in all 10 cases, marking specific time points for steroid pulse therapy, intravenous immunoglobulin (IVIG), and rituximab. Subsequently, we divided the cohort into two groups based on the use of intravenous immunoglobulin to plot both CK trends and cumulative prednisolone doses over the same observation period. Furthermore, a Kaplan–Meier curve for first remission was constructed, stratified by the interval from symptom onset to treatment initiation, and a log-rank test was used for comparative analysis. The initial and final statuses of functional muscle strength, as assessed by the Walton-Gardner-Medwin scale, were separately plotted for each group divided by the delay in treatment initiation. Statistical significance was set at *p* < 0.05. All analyses were conducted using STATA version 19 (Stata Statistical Software, Release 19. College Station, TX, USA: StataCorp LLC).

## 3. Results

### 3.1. Demographics and Clinical Characteristics

**Figure 1:**
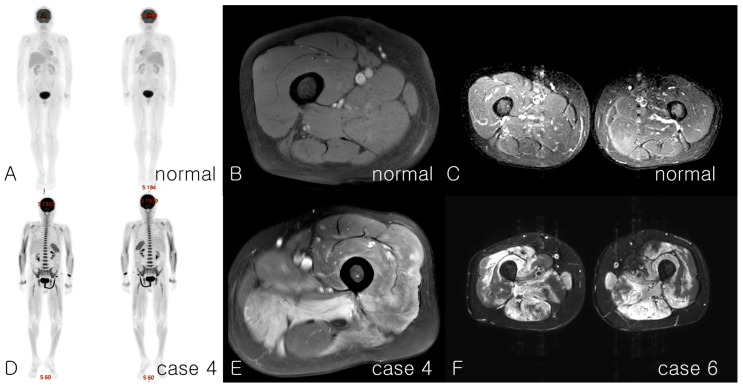
Characteristics of imaging findings in patients with anti-HMGCR-positive IMNM. (**A**), Positron emission tomography (PET) images of a normal case for comparison. (**B**), right thigh T1-weighted Dixon water only image; (**C**), both thigh T2-weighted short-tau inversion recovery (T2 STIR) image of a normal patient for comparison; (**D**), PET image of Case 4. Diffuse whole-body muscle showing increased signal, representing active inflammation; (**E**), MRI of the left thigh, T1 Spectral Presaturation with Inversion Recovery (SPIR) image of Case 4, adductor muscle edema, and inflammation relatively sparing the hamstring muscles. These MRI results were consistent with bilateral inflammatory changes similar to those observed in polymyositis.; (**F**), MRI of the lower leg, both thigh T2 STIR images, anterior rectus femoris, vastus intermedius, and posterior hamstring muscles showing extensive inflammatory and high-signal intensity of necrotizing myositis in Case 6. PET images are presented in the anterior and posterior views in sequence. IMNM, immune-mediated necrotizing myopathy; anti-HMGCR, anti-3-hydroxy-3-methylglutaryl-coenzyme A reductase; MRI, magnetic resonance imaging.

Ten patients (6 women and 4 men) were enrolled in the study (Table 2). All patients experienced progressive weakness in the proximal muscles, except for Case 10, who exhibited laboratory and imaging abnormalities indicative of myopathy but only subtle subjective symptoms. The median age at disease onset was 58 years (range, 33–86 years) (Table 2). The median time from symptom onset to confirmed diagnosis was 160 days (range, 38–650 days). The median follow-up period from presentation and treatment initiation was 722 days (range, 31–1552 days) and 687 days (range, 12–1510 days), respectively (Table 2). Eight patients were followed for more than 90 days after treatment. Nine of the ten patients had used statins before symptom onset, with a median statin exposure duration of 2 (range: 1–13) years, and the intensity of statin use was classified as low to high according to the American College of Cardiology (ACC)/American Heart Association (AHA) guidelines [18]. All patients had elevated CK levels ranging from 1438 to over 13,000 IU/L. In four patients, CK levels reached the maximum measurable limit, making it challenging to distinguish the condition from rhabdomyolysis. However, none of the patients presented with dark urine or acute renal failure. The mean ± SD of anti-HMGCR antibody was 183 ± 58.1 CU. Throughout the follow-up period, two patients (Cases 2 and 7) underwent repeated testing for HMGCR antibodies, consistently showing elevated levels similar to their initial values. All patients, except for Case 10, exhibited proximal muscle weakness in both the lower and upper limbs and experienced difficulties in climbing stairs and carrying heavy objects. One patient experienced respiratory distress (Case 4) that necessitated respiratory assistance during the acute recovery period. Electromyography revealed a diffuse myopathic pattern with frequent fibrillation and positive sharp waves. Complex repetitive discharges were observed in four patients. All patients underwent whole-body muscle MRI, except for one who underwent only a shoulder MRI, which revealed diffuse muscle edema and features resembling inflammatory myositis in the scanned area (Figure 1).

**Figure 2:**
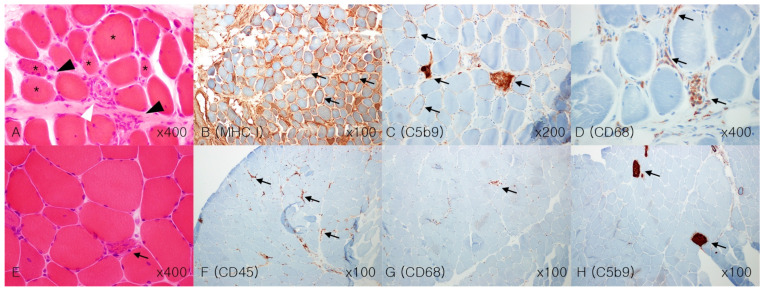
Muscle pathology findings in anti-HMGCR-positive IMNM patients. (**A**–**D**), Pathology of Case 1. Myopathic changes showing mild variation in myofiber size (asterisk) with multifocal myocyte degeneration (white arrowhead) and moderate chronic inflammatory cell infiltrates (black arrowhead), morphologically suggestive of inflammatory myopathy. Hematoxylin and eosin staining ((**A**), ×400). Immunohistochemical staining of MHC class I, diffuse increase (black arrow, (**B**), ×100) and C5b9 MAC presentation on degenerative muscle fibers (black arrow, (**C**), ×200). Immunohistochemical staining of CD68 in macrophages ((**D**), ×400); E–H, Pathology of Case 8. A few degenerating fibers with lymphocytic infiltration (black arrow, (**E**), ×400). Immunohistochemical staining of CD45, leukocyte common antigen, showed focal positivity in degenerating fibers (black arrow, (**F**), ×100), and CD68 macrophage antigen showed sparse macrophage infiltration (black arrow, (**G**), ×100), with focal positive degenerative fibers C5b9 MAC expression (black arrow, (**H**), ×100). The prominent deposition of MAC along the sarcolemma, along with the less prominent HLA class I expression, supports an antibody-mediated (humoral) process. Muscle cell necrosis and macrophage infiltration are characteristic features of muscle biopsy specimens obtained from patients with statin-associated autoimmune myopathy. IMNM, immune-mediated necrotizing myopathy; anti-HMGCR, anti-3-hydroxy-3-methylglutaryl-coenzyme A reductase; MAC, membrane attack complex; HLA, human leukocyte antigen.

Regarding autoantibody status, nine patients were tested for antinuclear antibodies (ANAs), and three showed ANA positivity with a speckled pattern. Seven patients underwent extractable nuclear antigen panel tests, and three showed positive for anti-SSA (Ro) antibodies. None of the patients tested positive for anti-Jo-1 antibodies. In addition to anti-HMGCR, seven patients were also tested for myositis-specific antibodies, which returned negative results (Table 2). Muscle biopsy pathology results indicated that the nine patients had necrotic muscle fibers at different stages, including muscle fiber size variability, suggesting regeneration after necrosis. Four patients showed the presence of the C5b9 membrane attack complex in the sarcolemma and sarcoplasmic reticulum. Five patients (56%) exhibited monomacrophage infiltration in the degenerative muscle fibers. Mild inflammatory changes were observed in six patients, with MHC class I, CD4, and CD8 cells in four, three, and three patients, respectively (Table 1 and Figure 2).

### 3.2. Treatment Regimens and Responses

**Figure 3:**
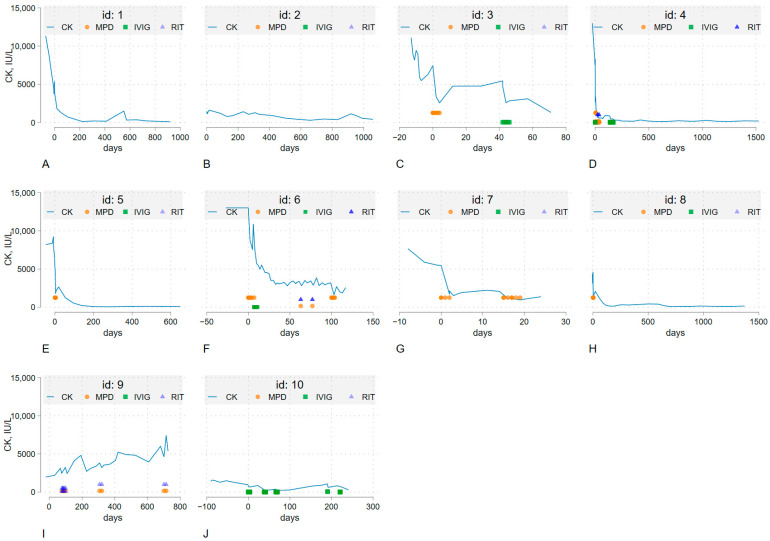
The trend in serum creatine kinase levels throughout the clinical course of all 10 cases is depicted (**A**–**J**). Day 0 represents the start of treatment. Each graph shares the y-axis. Methylprednisolone (MPD) pulse therapy, intravenous immunoglobulin (IVIG), and rituximab (RIT) were marked on the specified date if administered as part of the treatment regimen. Cases 1 and 2 achieved remission without the use of steroid pulse therapy but oral prednisolone (Table 2). Notably, cases 4, 6, and 9 received rituximab; however, two of these individuals (cases 6 and 9), who were under 40 years of age, exhibited a poor response to the treatment. In contrast, IVIG administration demonstrated a more favorable response in cases 3, 4, and 10. In comparison, Case 6, who received both IVIG and rituximab and was younger than 40, did not show such a good response. CK, creatinine kinase; IVIG, intravenous immunoglobulin; IU/L, international unit/L; MPD, methylprednisolone pulse therapy; RIT, rituximab.

**Figure 4:**
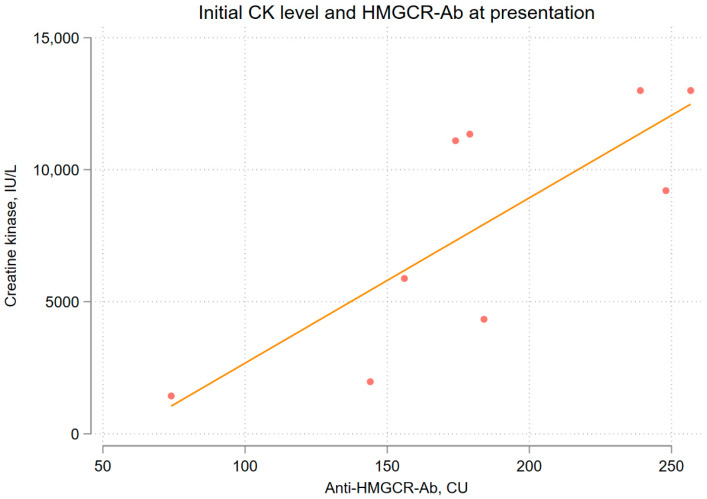
Correlation between initial serum CK levels and anti-HMGCR-Ab titer at presentation. The solid line indicates the fitted line (i.e., regression line). A positive correlation was observed between anti-HMGCR-Ab titer and initial serum CK levels. The correlation between anti-HMGCR antibody titer and initial serum CK level showed Spearman’s rho value of 0.7531 (*p* = 0.0028), indicating a positive linear relationship. Regression line is Y = 62.6 × X − 3576 (*p* = 0.0113, R^2^ = 0.624). Ab, antibody; CK, creatine kinase; CU, chemiluminescence unit; HMGCR, 3-hydroxy-3-methylglutaryl-coenzyme A reductase; IU/L, international unit/L.

**Figure 5:**
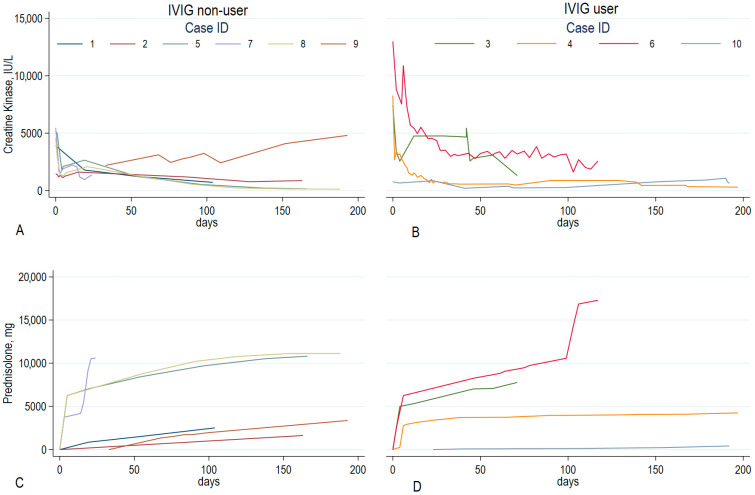
Comparison of creatinine kinase and cumulative prednisolone dose trends between IVIG users (**B**,**D**) and non-users (**A**,**C**). IVIG users presented with higher initial CK levels but tended to show a favorable response. All corticosteroid dosages were converted to prednisolone equivalent dosages and calculated as cumulative dosages on a given date. Day 0 represents the start of treatment. Each graph shares the y-axis. The cumulative prednisolone dosage curves rise sharply during the initial treatment phase, then either plateau or increase gradually over time. This pattern indicates the use of corticosteroid pulse therapy at the start of treatment, followed by a reduction to a maintenance dosage. However, Case 6 is an exception, as the patient is refractory to treatment and requires additional corticosteroid pulse therapy during the treatment course. CK, creatinine kinase; IVIG, intravenous immunoglobulin; IU/L, international unit/L.

During the initial treatment, seven patients underwent corticosteroid pulse therapy, and two were administered high-dose corticosteroids (1 mg/kg/day) without pulse therapy. These therapies were typically supplemented with additional immunosuppressive medications, such as methotrexate, azathioprine, tacrolimus, mycophenolate mofetil, rituximab, or intravenous immunoglobulin, except in two patients who were treated solely with prednisolone (Cases 1 and 5) (Table 2, Figure 3). Rituximab was administered to three patients (Cases 4, 6, 9), whereas IVIG was administered to four patients (Cases 3, 4, 6, 10) (Table 2, Figure 3). Among them, one patient was treated with IVIG with a reduced dosage of prednisolone (Case 10, 10 mg/day). Treatment strategies were tailored to each patient’s unique characteristics and the severity of their condition at the discretion of their physician. Most patients showed improvements in muscle strength, regardless of their serum CK levels. All patients experienced a decrease in CK levels after starting myositis treatment, except for Case 9, who was a 33-year-old woman without a history of statin use. Elevated CK levels persisted for at least three months. Initially, high CK levels fluctuated during the early treatment phase but usually decreased over 180 days after diagnosis (Figure 3). The percentages of patients who achieved normal CK levels at least once during each time interval from treatment initiation were 10%, 20%, 40%, 60%, and 60% on days 0–84, 85–180, 181–365, 366–730, and 731–1,370, respectively. Excluding Case 10, which was diagnosed as possible anti-HMGCR-positive IMNM, the correlation between anti-HMGCR antibody titer and initial serum CK level revealed Spearman’s rho value of 0.753 (*p* = 0.028), indicating a positive linear relationship (Figure 4). Among patients observed for over 180 days, six out of eight achieved complete remission, with a median of 242 days from treatment initiation, but three relapsed without statin re-challenges. When divided by IVIG administration, IVIG users were more likely to have higher initial CK levels and a better response of decreasing CK levels. Still, cumulative prednisolone doses were not different (Figure 5). Furthermore, delayed treatment initiation from symptom onset was correlated with a longer treatment time to achieve the first remission (Supplementary Figure S1). When divided by 180 days, the time span from symptom onset to treatment initiation, those who were treated within 180 days of symptom onset were more likely to achieve remission, as plotted on the Kaplan–Meier curve; however, this difference was not statistically significant due to the small number of patients (*p*-value = 0.12, Supplementary Figure S2). Regarding the functional outcomes assessed using the Walton-Garner-Medwin scale, most cases monitored for over 90 days exhibited favorable improvements, except for Case 6, a 36-year-old man who was refractory to IVIG, rituximab, and prednisolone pulse therapy (Figure 3, Supplementary Figure S3). In the early treatment group, 80% of the patients achieved normal muscle function. In contrast, in the delayed treatment group, where the interval from symptom onset to treatment initiation exceeded 180 days, none of the patients achieved normal muscle function. Two patients stopped following up, and one was still undergoing treatment in the early stages of the disease (Case 6, 117 days of treatment). A 33-year-old patient without clear statin exposure exhibited chronic active disease with high CK levels and no remission after 600 days, requiring prolonged immunosuppressive therapy and careful disease activity monitoring (Case 9). Both patients in Cases 6 and 9, who were under 40 years of age, exhibited a poor response to treatment with prednisolone pulse therapy, rituximab, or IVIG compared to other patients who were over 50 years of age.

## 4. Discussion

In this case series, we describe the clinical characteristics, treatment responses, and diagnostic challenges in patients with anti-HMGCR-positive IMNM. In 2010, the HMGCR antibody, initially referred to as the anti-200/100-kD antibody, was identified in patients who developed necrotizing myositis after using statins [19]. A subsequent study demonstrated that it targets the 97-kD HMGCR monomer and dimer [9]. As more research results have accumulated, anti-HMGCR-positive IMNM has been differentiated from anti-SRP-positive IMNM because of its specific characteristics and treatment response. Currently, IMNM is classified into three categories: anti-HMGCR-positive IMNM, anti-SRP-positive IMNM, and seronegative IMNM [3].

In the diagnostic criteria, the ENMC consensus suggests the classification of anti-HMGCR-positive IMNM without muscle biopsy pathology; however, the 2017 American College of Rheumatology (ACR)/ European League Against Rheumatism (EULAR) “classification criteria for adult and juvenile idiopathic inflammatory myopathies and their major subgroup scoring system” do not incorporate the criteria of anti-HMGCR or anti-SRP antibody but only anti-Jo-1 antibody, allowing classification as polymyositis and subgroups without IMNM specification when the score is ≥6.7 with pathology or ≥5.5 without pathology [15,20,21]. When applying the ACR classification of 2017 in our study of nine patients, excluding the case of possible anti-HMGCR-positive IMNM, six cases with pathology (66.7%) and seven cases without pathology (77.8%) were classified as “polymyositis, including IMNM subgroups” in comparison to the ENMC classification. It is recommended to employ classification criteria for the recruitment of patients in clinical studies to create homogeneous populations and thereby reduce bias. Clinically, the ENMC consensus is more suitable for practical purposes, facilitating prompt diagnosis and treatment of patients. The ACR/EULAR classification criteria may not be adequate for the recruitment of the IMNM subset for study purposes.

Accurately differentiating IMNM from other statin-associated muscle disorders, such as self-limited statin myopathy and rhabdomyolysis, is essential for proper diagnosis and effective management [22]. The presence of anti-HMGCR antibodies, a persistent elevation of serum CK levels for several months despite discontinuation of statin use, and profound muscle weakness are characteristic of anti-HMGCR-positive IMNM, as these features are absent in patients with self-limited statin-induced myopathy or rhabdomyolysis [23]. Weakness in IMNM significantly affects daily activities and may be accompanied by dysphagia [8]. In our patients, even with definitive treatment, CK levels approached normal after six months, and most of them had disabilities in daily activities initially.

Statin-associated muscle symptoms are the most commonly reported side effects [24]. Monitoring patients for indications of muscle pain, tenderness, or weakness is recommended, particularly during the initial months of statin therapy and after subsequent dose escalation [22]. CK measurements should be performed when symptoms are present. Statin treatment should be promptly discontinued if elevated CK levels are detected (i.e., CK levels > 10 times the upper limit of normal) or if myopathy is suspected. In cases of a moderate increase in CK levels (i.e., 3–10 times the upper limit of normal), CK levels should be monitored weekly, and specialist advice should be sought [5,22,25].

Some patients with myositis are initially diagnosed with abnormal liver function test results in blood tests; therefore, they often visit a gastroenterologist in the early stages of the disease. However, testing for HMGCR antibodies is not readily available to physicians who encounter patients with persistently abnormal liver enzyme levels, even after discontinuing statin therapy. Another significant factor contributing to this underestimation is that many physicians are unaware that most commercial myositis-specific antigen panel test kits do not test for HMGCR antibodies but instead include SRP antibodies. Anti-HMGCR antibodies were identified and introduced into clinical practice later than anti-SRP antibodies. In this study, delayed treatment initiation from symptom onset was correlated with a longer treatment time required to achieve the first remission. Notably, two patients under the age of 40 were unable to achieve remission, regardless of the delay in treatment initiation or treatment regimen, which included IVIG or rituximab.

As demonstrated in our Case 9, although she developed anti-HMGCR-positive IMNM, the association with statins sometimes remains unclear; younger patients who have not been exposed to statins also develop this disease. Various foods and microorganisms, including mushrooms, fungi, and bacteria, contain statin-like molecules that lower cholesterol levels in humans [3]. It is suggested that the uptake of these substances is a stimulus for susceptible individuals [8,26]. Case 9 may be attributed to these environmental factors combined with individual vulnerability rather than being caused by statin medication.

Despite the tendency of HMGCR antibody levels to decrease following myositis treatment and statin discontinuation, the antibodies remain detectable. In contrast, in patients without a history of statin use, antibody levels rarely decrease, even after treatment [10]. In this study, two patients, both statin users over the age of 50 years, exhibited no significant changes in the titers of repeated measurements of anti-HMGCR antibodies. However, the titer change and prognosis have not been clearly investigated. A higher titer of non-statin-associated anti-HMGCR-positive IMNM is typically associated with a younger age and a poorer response to therapy [27]. The pathological toxicity of this antibody has been elucidated through animal experiments, in vitro studies, and clinical investigations, which have demonstrated a positive correlation with CK levels, as in our results [10]. The persistence of HMGCR antibodies in the body suggests that muscle fiber destruction and regeneration are ongoing. Therefore, even if muscle strength improves in some patients, their CK levels may not return to normal values. Treatment goals should be flexible, considering the patient’s potential for muscle recovery [28].

In our study, one patient exhibited a facial rash resembling a lupus malar rash and tested positive for ANA with a speckled pattern and anti-SSA (Ro) antibodies. However, this was insufficient for classifying systemic lupus erythematosus or Sjögren’s syndrome. Joseph et al. reported a similar case of a skin rash akin to dermatomyositis in 19 patients with anti-HMGCR-positive IMNM [29]. Their study reported a higher association with statin exposure in 16 of 19 cases (84%), comparable to our series, which showed an association in nine of the ten cases (90%). Other studies have reported that statin use was associated with 15% to 65% in anti-HMGCR-positive IMNM cohorts [9]. In individuals aged > 50 years, the history of statin exposure is 90% [3,19]. In addition, we observed one case of dysphagia during follow-up, a condition that accompanies anti-HMGCR-positive IMNM in 20–50% of patients in large cohorts [8]. One patient presented with acute respiratory arrest, a rare occurrence associated with IMNM, underscoring the necessity for vigilance in this disease entity, as early treatment should focus on immunomodulation [30]. Among the two patients aged < 50 years, a 36-year-old man was in the acute phase of the disease, with MRI revealing extensive muscle necrosis. Another patient, a 33-year-old woman with no history of statin use, exhibited chronic active disease with persistently elevated CK levels despite treatment with various immunosuppressive agents, including rituximab. Younger age and lack of history of statin exposure are associated with poor prognoses [3,31]. A multivariate analysis from another study revealed that younger age is a significant poor prognostic factor, whereas statin exposure status is not [32].

In terms of treatment, corticosteroids remain the primary option, but recently, IVIG has emerged as a promising choice for reducing prednisolone use and even achieving a steroid-free regimen [16,33,34]. Meyer et al. reported that out of 55 patients, 14 (25%) achieved remission successfully without the use of corticosteroids. Of these, 7 (50%) were treated with a combination of IVIG and a steroid-sparing immunosuppressant (SSI), while the other 7 received only SSI. In the group receiving corticosteroid-based treatment, the majority of patients (37 out of 41) achieved remission with either a dual steroid/SSI or triple steroid/SSI/IVIG regimen. The remaining four patients did not initially respond to the triple therapy (corticosteroid, IVIG, SSI) but eventually achieved remission through switching or step-up strategies [16]. However, the study was retrospective, with 40% of the 55 patients exhibiting normal strength at the time of diagnosis, and none were younger than 40 years old. Among those who commenced treatment earlier without muscle weakness, 56% (5 of 9) were more likely to be managed without steroids, whereas only 8% (1 of 13) of the delayed treatment group were managed without steroids. The author emphasized the importance of initiating treatment early, noting that an initially milder form of the disease can eventually lead to muscle weakness and a poor response to treatment. These findings suggest that careful observation of asymptomatic patients is warranted, and that treatment may be considered for patients with mild symptoms if MRI STIR imaging shows findings suggestive of muscle inflammation.

In our study, due to the limitation of a small patient sample, we were unable to confirm the favorable outcomes statistically; however, we did find a correlation between the time to achieve remission and the interval from symptom onset to treatment initiation. In relation to IVIG therapy, the clinical course of serum CK levels indicated a better treatment response. The cumulative prednisolone treatment dosages were not different, as three out of four patients who received IVIG also underwent corticosteroid pulse therapy. Notably, without steroid pulse therapy, Case 10, who received IVIG and a daily low dose of prednisolone (10 mg/day), could achieve remission but experienced recurrent relapses that were managed with IVIG again. However, Case 6, who was younger than 40, showed less favorable response to treatment despite IVIG, steroid pulse therapy, and rituximab. Rituximab is typically not used as a first-line treatment for anti-HMGCR-positive IMNM, unlike IVIG, but it is utilized for anti-SRP-positive IMNM. Nevertheless, in cases that are resistant to other therapies, rituximab becomes necessary, with a 43% response rate reported in a different study [35]. Also, in our Case 9, a non-statin user aged < 40 years, there was no favorable response, and active inflammation persisted with CK levels exceeding 5000 IU/L. In both Cases 6 and 9, a common factor was the young age of less than 40, among several possible factors associated with a poor response to therapy, such as receiving no more than one IVIG administration, longer symptom duration, or a history of non-statin exposure [16,31,32].

This study underscores the complexity and clinical significance of anti-HMGCR-positive IMNM. This case series underscores the importance of distinguishing IMNM from benign statin-induced muscle disorders to ensure timely diagnosis and treatment of IMNM. Elevated CK levels, muscle biopsies revealing necrosis and regeneration, and the presence of anti-HMGCR antibodies serve as pivotal diagnostic markers [15]. Despite advancements in treatment strategies, the persistence of disease flares and chronic activity in some patients signals the challenge of achieving complete remission and maintaining long-term disease stability. Multidisciplinary collaboration and vigilance are essential for this new myositis entity.

In conclusion, the prognosis of anti-HMGCR-positive IMNM is generally less favorable when treatment is delayed after symptom onset. Further research is necessary to elucidate the risk factors associated with poor prognosis and inform decisions regarding appropriate treatment regimens.

## Figures and Tables

**Table 1:** Walton-Gardner-Medwin Scale [17].

Grade	
0	HyperCkemia, all activities normal
1	Normal gait, unable to run freely. Myalgia
2	Difficulty walking on tiptoes. Waddling gait
3	Evident muscular weakness. Steppage and climbing stairs with a banister
4	Difficulty to rise from the floor, Gowers’ sign
5	Inability to rise from the floor
6	Inability to climb stairs
7	Inability to rise from a chair
8	Unable to walk without assistance
9	Unable to eat, drink, or sit without assistance

**Table 2:** Demographics, clinical characteristics, and treatment responses of patients.

CASE ID	1	2	3	4	5	6	7	8	9	10	Mean ± SD (Median) or N (%)
Sex	M	F	F	M	F	M	M	F	F	F	Women: 6 (60)
Age	79	61	51	58	58	36	86	54	33	73	58.0 ± 17.1 (58)
Statin use	Yes	Yes	Yes	Yes	Yes	Yes	Yes	Yes	No	Yes	9 (90)
Duration of statin administration	2 years	8 years	Unknown	1.5 years	3 years	1 year	2 years	Unknown	NA	13	4.4 ± 4.5 (2)
Statins	Atorvastatin, rosuvastatin	Atorvastatin	Unknown	Atorvastatin, rosuvastatin	Atorvastatin	Atorvastatin	Atorvastatin	Unknown	NA	Atorvastatin	
Dose	High intensity	Moderate intensity	Unknown	Moderate intensity	Unknown	Moderate intensity	Low intensity	Unknown	NA	Low intensity	
Muscle involvement pattern	Proximal, upper, and lower extremities	Proximal, upper, and lower extremities	Proximal, upper, and lower extremities	Proximal, upper, and lower extremities, Respiratory distress	Proximal, upper, and lower extremities	Proximal, upper, and lower extremities	Proximal, upper, and lower extremities	Proximal, upper, and lower extremities	Proximal, upper, and lower extremities	Not definite	
MRC Muscle Grade (0–5)											
Arm raise	5	4	4	3	4.5	4	3	4	4	5	4.1 ± 0.7 (4)
Leg raise	5	4	4	2	4.5	4	3	4	4	5	3.9 ± 0.9 (4)
Walton-Gardner-Medwin scale, initial→ at last	8 → 1	3 → 1	7 → 3	9 → 0	6 → 0	6 → 4	8 → 8	6 → 0	3 → 1	1 → 0	Initial: 5.7 ± 2.6 (6) Last: 1.8 ± 2.6 (1)
Skin lesion	None	None	None	None	None	None	None	Face	None	None	
HMGCR Ab, CU (ref. <20 CU)	179	74	174	239	248	256.7	156	184	144	202	183 ± 58.1 (179)
Initial CK, IU/L (ref. women 32-135, men 44-245 IU/L)	11,351	1438	11,101	13,000	9209	13,000	5883	4339	1977	1569	7922 ± 4602 (9209)
Maximum, CK, IU/L	11,351	14,156	11,101	13,000	9209	13,000	7659	4597	13,254	1569	
Minimum, CK, IU/L	73	278	1307	101	47	2977	949	68	2193	202	
ANA	Negative	Negative	Negative	Negative	ANA 1:160, Speckled	ND	Negative	ANA 1:640, Speckled	ANA 1:160, Speckled	Negative	3 (30)
ENA	ND	ND	Negative	Ro +	Ro +	Negative	Negative	Ro +	Negative	ND	
Other MSA ^a^	ND	ND	ALL negative	ALL negative	ND	ALL negative	ALL negative	ALL negative	ALL negative	All negative	
Inflammatory marker	Normal	Normal	Normal	Normal	Normal	Normal	Normal	Normal	Normal	Normal	
MRI, involved muscles	Whole-body muscle: proximal and distal, upper and lower limbs	Only shoulder MRI checked: Proximal Arm, chest, back, neck	Whole-body muscle: proximal and distal, arm, leg, back	Diffuse upper and lower limbs	Proximal upper and lower	Proximal, upper and lower limbs, back	Whole-body muscle: proximal and distal, upper and lower limbs, back and chest	Proximal, upper and lower limbs, back	Whole-body muscle	Subtle myopathic change, upper and lower limbs	
EMG	Generalized myopathy	Generalized myopathy	Generalized myopathy	Generalized myopathy	Generalized myopathy	Generalized myopathy	Generalized myopathy	Generalized myopathy	Generalized myopathy	ND	
Muscle biopsy										ND	
Muscle fiber size variability	Yes	Yes	Yes	Yes	Yes	Yes	Yes	Yes	Yes	NA	
Muscle inflammation	Yes	No	No	Yes	No	Yes	Yes	Yes	Yes	NA	
MHC Class I expression	Yes	No	No	No	No	Yes	Yes	Yes	No	NA	
C5b-9, MAC expression	Yes	No	No	No	No	Yes	Yes	Yes	No	NA	
CD4/CD8 cells	No/No	No/No	No/No	No/No	No/No	Yes/Yes	Yes/Yes	Yes/Yes	No/No	NA	
Monocyte, macrophage	Yes	No	No	No	No	Yes	Yes	Yes	Yes	NA	
Time interval, days											
Interval, symptom to diagnosis	170	614	363	54	84	43	166	155	650	38	233.7 ± 230.6 (160)
Interval ^b^, symptom to treatment	189	474	323	38	84	47	154	141	657	90	255.4 ± 233.5 (166)
Interval ^c^, to first remission	223	660	NA	245	138	NA	NA	651	NA	41	353.2 ± 213.6 (242)
Total follow-up ^d^	988	1060	84	1552	697	144	31	1377	747	331	701 ± 544 (722)
Treatment follow-up time ^e^, days	917	1060	71 (FU loss)	1510	649	117	12 (FU loss)	1372	725	293	670 ± 548 (687)
Referred during myositis treatment		Yes		Yes					Yes		
Medication											
Prednisolone, pulse therapy (dose, mg)	No	No	Yes (1000)	Yes (1000)	Yes (1000)	Yes (1000)	Yes (1000)	Yes (1000)	Yes (250)	No	7 (70)
Initial Prednisolone dose, mg/day	45	50	1000	1000	1000	1000	1000	1000	250	10	
Other drugs	No	MTX, azathioprine	IVIG	MTX, IVIG, rituximab	No	MTX, IVIG, rituximab	MTX	MTX	MTX, azathioprine, tacrolimus, rituximab	MTX IVIG	MTX: 7 (70) IVIG: 4 (40)
Treatment response											
Current status	Recurrent, in remission	Recurrent, in remission	Follow-up loss (during active phase)	In remission	In remission	Active	Follow-up loss (during active phase)	In remission	Chronic active	Recurrent, in remission	6 (60) achieved remission
Disease pattern	Polycyclic	Polycyclic	NA	Unicyclic	Unicyclic	NA	NA	Unicyclic	Chronic	Polycyclic	
Walton-Gardner-Medwin scale, last	1	1	3	0	0	4	8	0	1	0	1.8 ± 2.6 (1)
Dysphagia	Yes	No	No	No	No	No	No	No	No	No	1 (10)
Current dyslipidemia management	Ezetimibe 10 mg + Omega-3, 1 g	Ezetimibe 10 mg	None	Ezetimibe 10 mg, PCSK9 inhibitor	None	None	Ezetimibe	Ezetimibe 10 mg, omega-3, 2 g	None	Ezetimibe 10 mg	

^a^ Assessment of myositis-specific antibodies (MSAs) was conducted using a comprehensive panel, which included testing for anti-JO-1, PL-7, PL-12, EJ, OJ, SRP, MI2-alpha, MI2-beta, MDA5, TIF1-gamma, and NXP-2 antibodies in serum. ^b^ From the time symptoms started to the time treatment began. ^c^ From the time treatment started to the first time in remission (if possible). ^d^ From the presentation to the last follow-up date. ^e^ From the time treatment was started to the last follow-up date. ANA, antinuclear antibody; CU, chemiluminescence unit; ENA, extractable nuclear antigen test; FU, follow-up; HMGCR Ab, 3-hydroxy-3-methylglutaryl-coenzyme A reductase antibody; MAC, membrane attack complex; MHC, major histocompatibility complex; MRC, Medical Research Council muscle strength scale; MSA, myositis-specific antibody; MTX, methotrexate; NA, not applicable; ND, not done; PCSK9 inhibitor, proprotein convertase subtilisin kexin 9 inhibitor; ref, reference.

## Data Availability

The original contributions presented in this study are included in the article and Supplementary Material. Further inquiries can be directed to the corresponding author(s).

## Supplementary Materials

The following supporting information can be downloaded at: https://www.mdpi.com/article/10.3390/jcm14186610/s1, Figure S1: Correlation between the duration from symptom onset to the initiation of treatment and the time required to achieve the first remission; Figure S2: Kaplan-Meier survival estimates for first remission achievement; Figure S3: Change of Walton-Gardner-Medwin score.

## Abbreviations

The following abbreviations are used in this manuscript:

ANA	Antinuclear antibodies
CK	Creatine kinase
ENMC	European Neuromuscular Centre
HLA	Human leukocyte antigen
HMGCR	3-hydroxy-3-methylglutaryl-coenzyme A reductase
IMNM	Immune-mediated necrotizing myopathy
IVIG	Intravenous Immunoglobulin
MAC	Membrane attack complex
MHC	Major histocompatibility complex
PCSK9	Proprotein convertase subtilisin kexin 9
SRP	Signal recognition particle
SSI	Steroid-sparing immunosuppressant
