# Supplementary material for: Clinical Characteristics and Management of Statin-Associated Anti-3-Hydroxy-3-Methylglutaryl-Coenzyme A Reductase Immune-Mediated Necrotizing Myopathy
Source: J Clin Med. 2025 Sep 19;14(18):6610. doi: 10.3390/jcm14186610 (PMC12471232; doi:10.3390/jcm14186610)
Supplement: Supplementary file 1 [file jcm-14-06610-s001.zip › jcm-3813394-supplementary.pdf]

Supplementary Materials

J Yoon, SW Kim, SH Kim, et al. Clinical Characteristics and Management of Statin Associated Anti-3-hydroxy-3-methylglutaryl-coenzyme A reductase Immune-Mediated Necrotizing Myopathy

Contents

Supplementary Figure S1. Correlation between the duration from symptom onset to the initiation of treatment and the time required to achieve the first remission. 2

Supplementary Figure S2. Kaplan-Meier survival estimates for first remission achievement. 3

Supplementary Figure S3. Change of Walton-Gardner-Medwin score 4

Supplementary Figure S1. Correlation between the duration from symptom onset to the initiation of treatment and the time required to achieve the first remission

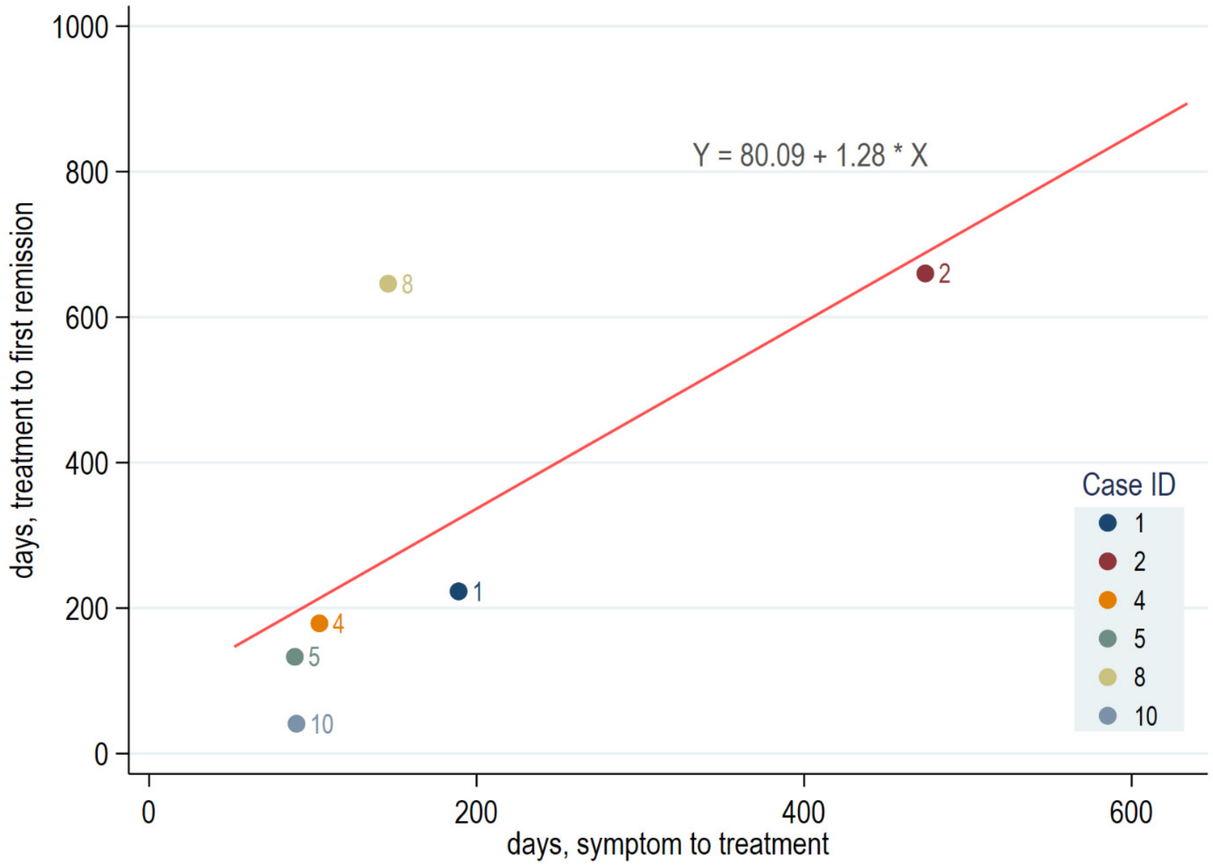

A significant correlation was identified between the duration from symptom onset to the initiation of treatment and the time required to achieve the first remission (Spearman's Rho 0.886, P-value 0.0259). A significant correlation was identified between the duration from symptom onset to the initiation of treatment and the time required to achieve the first remission (Spearman's Rho 0.886, P-value 0.0259). This demonstrates that the commencement of treatment at a later stage is associated with a prolonged treatment necessary to achieve remission, if possible (six out of ten experienced first remission regardless of relapse thereafter).

Supplementary Figure S2. Kaplan-Meier survival estimates for first remission achievement.

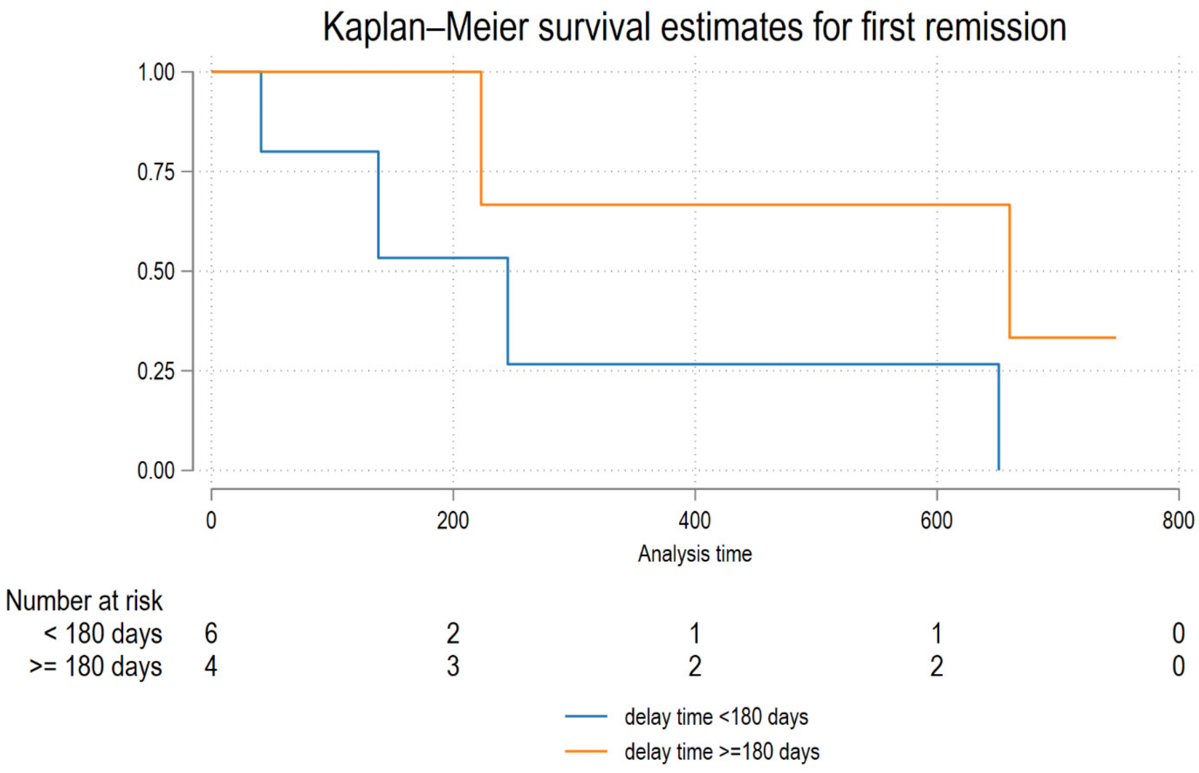

Kaplan-Meier survival estimates for first remission achievement.
When categorized by the delay in time to treatment from symptom onset, patients who received treatment within 180 days were more likely to achieve remission, although this finding was not statistically significant (Log rank test, P-value 0.12).

Supplementary Figure S3. Change of Walton-Gardner-Medwin score

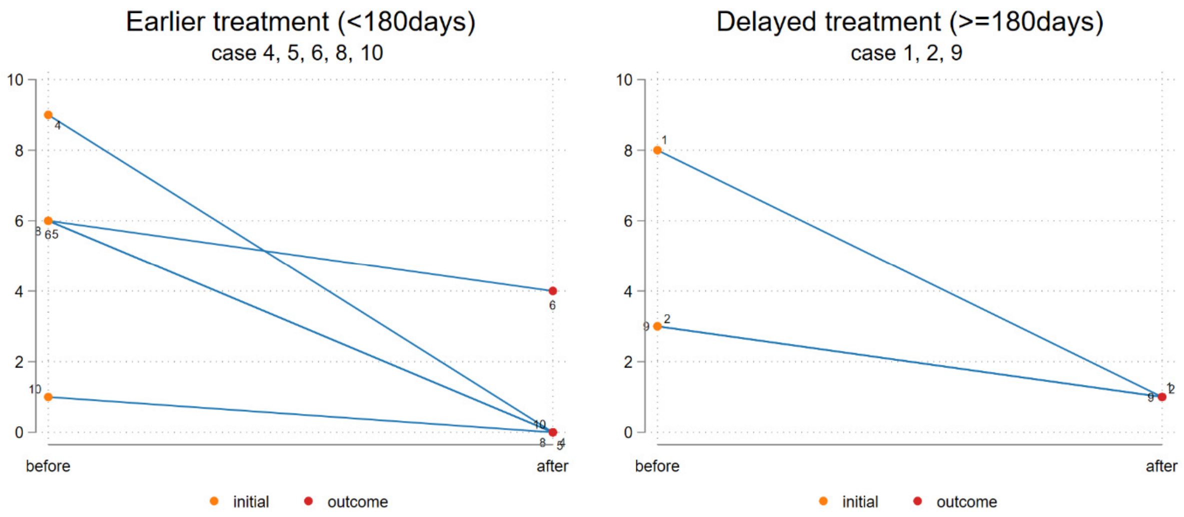

Change of Walton-Gardner score from the initial to the final assessment in cases with follow-up periods exceeding 90 days post-treatment. It should be noted that Cases 3 and 7 were excluded due to loss to follow-up. In the early treatment group, 80% achieved normal muscle function, whereas in the delayed treatment group, where the interval from symptom onset to treatment initiation exceeded 180 days, none achieved normal muscle function
